# Computational Properties of the Hippocampus Increase the Efficiency of Goal-Directed Foraging through Hierarchical Reinforcement Learning

**DOI:** 10.3389/fncom.2016.00128

**Published:** 2016-12-12

**Authors:** Eric Chalmers, Artur Luczak, Aaron J. Gruber

**Affiliations:** Department of Neuroscience, University of LethbridgeLethbridge, AB, Canada

**Keywords:** reinforcement learning, hierarchical learning, hippocampus, planning, context

## Abstract

The mammalian brain is thought to use a version of Model-based Reinforcement Learning (MBRL) to guide “goal-directed” behavior, wherein animals consider goals and make plans to acquire desired outcomes. However, conventional MBRL algorithms do not fully explain animals' ability to rapidly adapt to environmental changes, or learn multiple complex tasks. They also require extensive computation, suggesting that goal-directed behavior is cognitively expensive. We propose here that key features of processing in the hippocampus support a flexible MBRL mechanism for spatial navigation that is computationally efficient and can adapt quickly to change. We investigate this idea by implementing a computational MBRL framework that incorporates features inspired by computational properties of the hippocampus: a hierarchical representation of space, “forward sweeps” through future spatial trajectories, and context-driven remapping of place cells. We find that a hierarchical abstraction of space greatly reduces the computational load (mental effort) required for adaptation to changing environmental conditions, and allows efficient scaling to large problems. It also allows abstract knowledge gained at high levels to guide adaptation to new obstacles. Moreover, a context-driven remapping mechanism allows learning and memory of multiple tasks. Simulating dorsal or ventral hippocampal lesions in our computational framework qualitatively reproduces behavioral deficits observed in rodents with analogous lesions. The framework may thus embody key features of how the brain organizes model-based RL to efficiently solve navigation and other difficult tasks.

## Introduction

Reinforcement Learning (RL) provides a computational account of how an agent can learn appropriate behavior by interacting with its environment, discovering through experience what actions lead to rewards or punishments, and how to maximize the sum of future rewards (Doya, 2007). The mammalian brain is thought to employ a form of RL using information about reinforcements encoded by dopamine neurons (Montague et al., 1996; Reynolds et al., 2001; Samejima et al., 2005; Daw et al., 2006).

RL algorithms estimate the value associated with each action possible from a particular state in the environment. If the agent is in state *s*, and executing action *a* will bring it to a new state *s*′, then the value *Q* of executing action *a* is expressed as:

(1) $$Q(s,a)=\text{E}[r+\gamma \times {\text{max}}_{a\prime }(Q(s\prime ,a\prime ))]$$

Where *r* is the expected reward associated with executing action *a* from state *s*, and the *discount factor* γ ϵ [0, 1] controls the agent's degree of preference for immediate rewards over future ones. According to this recursive equation, the value of executing action *a* from state *s* is a discounted sum of all future rewards following that action. RL algorithms incrementally update these value estimates in response to errors between the expected and observed reinforcements. Q-learning and SARSA (Sutton and Barto, 1998) are two “model-free” algorithms for reinforcement learning: these algorithms rely on *Q* (*s, a*) action value estimates to make decisions, and update them in response to reward prediction errors. Model-free RL is associated with habitual learning (Daw, 2012).

In the present work we focus on “Model-based” RL (MBRL) algorithms, which additionally build an internal model of the agent's world through experience. The model learns transition probabilities P(*s*′|*s, a*) describing how actions lead from one state to another, and the reward function *r* = R (*s, a, s*′) which specifies the reward associated with each transition. Value estimates can be calculated using the model:

(2) $$Q\left(s,a\right)=\sum_{s\prime }P\left(s,a,s\prime \right)\left(\text{R}\left(s,a,s\prime \right)+\gamma {\text{max}}_{a\prime }Q\left[s\prime ,a\prime \right]\right)$$

MBRL is associated with *goal-directed* planning, wherein artificial or biological agents use knowledge of action outcomes to flexibly make choices (Daw, 2012; Botvinick and Weinstein, 2014). The agent's world model captures the learned dynamics of the world and allows knowledge gained in one place to immediately become part of decision-making elsewhere: thus the agent can effectively “look ahead” during decision making.

However, conventional MBRL algorithms involve a large computational overhead, especially if the task being learned involves many states: after each new experience, Equation (2) must be repeatedly evaluated in order to propagate new information throughout all the model's *Q* (*s, a*) value estimates. This suggests that model-based learning and behavior are cognitively demanding, and prompted research into potential brain mechanisms for reducing the cognitive burden (Daw et al., 2005; Cushman and Morris, 2015). Furthermore, MBRL algorithms do not explain the ease with which animals adapt to environmental changes, particularly in spatial navigation (McDonald et al., 2002; Roberts et al., 2007), suggesting that animals' implementation of model-based learning includes features absent from current MBRL algorithms.

**Figure 1:**
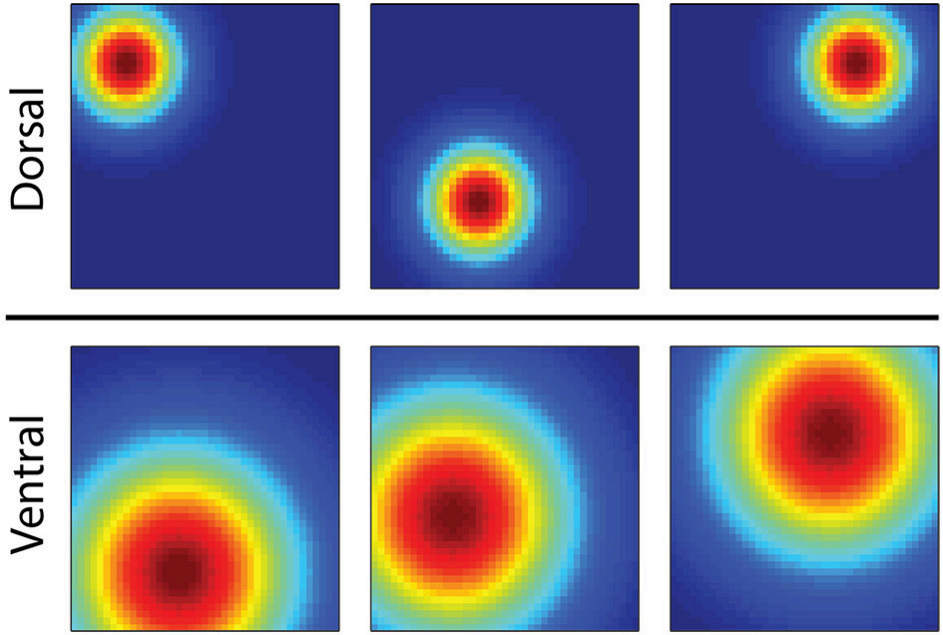
**Illustration showing the firing fields of six hippocampal place cells of an animal in a square enclosure**. Red encodes locations of rapid action potential firing, and blue encodes no firing. The three simulated cells in the dorsal hippocampus activate in distinct regions of the box, while those in the ventral hippocampus activate across a larger area.

Here we consider the possibility that several computational properties of the hippocampus serve to endow the brain's MBRL system with both flexibility and computational efficiency. The first is exhibited by the unique operation of *Place cells* in the hippocampus, which activate in particular regions of an environment, exhibit spatial specificity which changes in a gradient along the septotemporal axis of the hippocampus (Strange et al., 2014). Place cells in the dorsal hippocampus represent small regions while those in the ventral hippocampus represent larger regions (Figure 1). It is speculated that this gradient of spatial specificity is linked to a similar gradient in the spatial firing of entorhinal grid cells (Strange et al., 2014) from which place cells likely receive input (Moser et al., 2015). At choice points in a maze, the dorsal hippocampus generates so called “*forward sweeps*” of activity encoding possible future trajectories from the current location (Johnson and Redish, 2007; Pfeiffer and Foster, 2013), possibly indicating an active process of predicting consequences and planning paths to goals (Johnson and Redish, 2007).

Moreover, the hippocampus is associated with representing the current context, which we consider here to be the gestalt state of the environment that influences the set of actions (or policy) the animal has learned to engage (McDonald et al., 2002; Gruber and McDonald, 2012). Context changes can trigger a *global remapping* phenomenon (Jezek et al., 2011) in which a new set of place cells are recruited to represent the new context.

We hypothesize that the hierarchical representation of spatial state combined with forward sweep planning and context representation, could support hierarchical MBRL in the spatial domain for efficient and flexible foraging. We tested this by constructing and evaluating a novel computational RL framework that includes similar features. We implement the framework in an abstract way, trading biological accuracy for ease of illustrating its computational properties.

Several interesting properties emerge from this framework. Model-based learning, planning, and goal-setting are more efficient at the higher, more abstract levels of the hierarchy because they generalize over large areas. On the other hand, the lowest levels of the spatial hierarchy represent the world with the most spatial detail, and are best suited to creating plans (trajectories) to achieve high-level goals. This planning is inspired by the forward sweep phenomenon. Lastly, the framework provides a means of learning several policies (tasks) associated with different contexts, and switching rapidly between them; an ability exhibited by animals, but not by conventional MBRL algorithms. Our new hierarchal MBRL framework is thus highly flexible and efficient, and provides a normative explanation for several features of processing in the hippocampus.

## Methods

### Computational model of place-cell-supported reinforcement learning

In this section we propose a hierarchical learning system in which a task is represented at multiple levels of abstraction, ranging from very detailed to very general. Model-based reinforcement learning occurs at each level of abstraction, such that high-level models learn general strategies (i.e., “move toward the back of the maze”) while low-level models learn the details required for efficient navigation (i.e., “the doorway on the right leads to a hallway that leads to the back of the maze”). As we will show, the addition of these abstract levels increases efficiency by relieving lower-level world models of much of the computational burden of model-based RL. The remainder of this section provides details of the hierarchical learning scheme. For details of how learning mechanisms at various levels interact, see Section: “Hierarchical Planning Architecture”.

**Figure 2:**
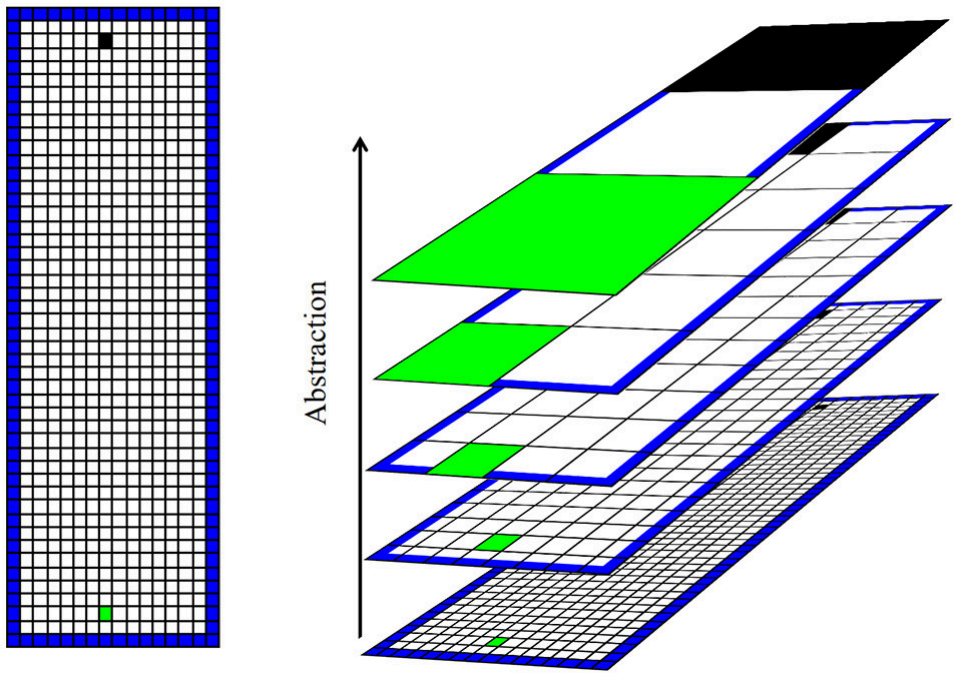
**Illustration of the task and spatial hierarchy. Left:** illustration of the simple simulated “open field” learning task. The artificial agent (initial position marked in black) must learn to navigate to the goal (marked in green) in a rectangular area. The open arena is divided into a grid of states. The agent is aware only of its current state, and cannot sense (e.g., see or smell) the goal. **Right**: hierarchical representation of space used by the agent. Higher levels of abstraction aggregate states into progressively larger macro-states. The arena is represented by only a few states at high levels of abstraction.

We consider a simple spatial navigation task, which simulates an open arena divided into a grid of discrete states that an agent can occupy. We create several levels of abstraction with progressively coarser divisions of space by recursively aggregating groups of four adjoining states (Figure 2). The result is a spatial hierarchy roughly analogous to that in the hippocampus. One model-based RL algorithm runs at each level, learning the action sequence required at its level to reach the goal. Due to the coarse spatial representation at higher levels, these learn to set general spatial goals, whereas the lower levels plan a state sequence (e.g., route) to the goal at successively finer spatial resolutions. We refer to this as a “framework” rather than a “model” in order to avoid confusion with the model of the environment employed within the MBRL algorithm.

The hierarchical scheme illustrated in Figure 2 is not intended to accurately model place cell firing fields, but rather to implement the general concept of hierarchical spatial representation. In reality, place fields overlap such that an ensemble of ventral hippocampal place cells could precisely and accurately encode spatial state (Keinath et al., 2014). In this work we focus on the fact that spatial scale increases along the septotemporal axis of the hippocampus and explore the computational benefits this provides. Our framework also abstracts away the roles of various other RL-related brain structures that interact with the hippocampus. For example, value information encoded in the striatum (Samejima et al., 2005) and neocortex (Gruber et al., 2010; Seo et al., 2014) will here be encoded along with spatial state. The medial pre-frontal cortex receives projections from the ventral hippocampus and is associated with task abstraction and prospection (Buckner and Carroll, 2007; Passingham and Wise, 2014). The state, value, abstraction, and planning-related information distributed among various brain structures and circuits, is encapsulated here in a single computational framework. This allows an easier exploration of the computational properties of the framework in the absence of assumptions and dynamical considerations required for connectionist models.

All model-based RL algorithms used in our experiments were tabular, meaning that a table T [*s, a, s*′] tracked the number of times action *a* from state *s* resulted in a transition to *s*′. Transition probabilities *P* (*s*′|*s, a*) may be estimated using this table:

(3) $$P\left(s\prime \text{|}s,a\right)\;=\;\frac{\text{T}\left[s,a,s\prime \right]\;+\;c}{\sum_{s_{2}}\left(\text{T}\left[s,a,s_{2}\right]+c\right)}$$

where *c* is a prior count which we set to one. A second table R [*s, a, s*′] tracked expected rewards associated with each transition. Upon executing action *a* from state *s* and transitioning to state *s*′ with reward *r*, the table was updated as follows:

(4) R [s,a,s′] = R [s,a,s′] + (r−R [s,a,s′])/T [s,a,s′]

making R [*s, a, s*′] a running average of the rewards experienced during the <*s, a, s*′> transition.

After each action, *Q*-value estimates were computed using Equation (2) and the prioritized sweeping approach described by Moore and Atkeson (1993). Prioritized sweeping updates value estimates in response to new information, but forgoes these updates when the information is not new. We speculate that the reactivation of trajectories in the hippocampus after reward (Singer and Frank, 2009) and during rest (Wilson and McNaughton, 1994) could support such a selective updating mechanism. The prioritized sweeping algorithm allows an agent who has learned the task completely to shift into a model-free mode wherein it relies on cached action values for decision making. It shifts back to the model-based mode when unexpected state transitions or values are encountered. This shift from model-based to model-free paradigms may correspond to the change from goal-directed to habitual behavior in rats as learning progresses (Packard and McGaugh, 1996).

Discount factors (which control the extent to which immediate rewards are favored over distant ones) at each level of the hierarchical algorithm were tuned in the range of 0.5–0.9, with higher levels having lower discount factors (higher preference for immediate reward). This tuning seems important to prevent any one level from dominating the goal-setting process. The tuning was done empirically for good performance in an open arena navigation task, and held fixed at the same values for all subsequent testing. Each model-based RL algorithm learns the task (i.e., creates value maps of the space) independently, at its own level of discounting and at its own spatial scale.

Models at each level of abstraction were updated according to Equations (3, 4) and the prioritized sweeping algorithm each time the agent's movement constituted a state transition *at that level*. At abstract levels, the *r* value used in Equation (4) was the maximum reward experienced while the agent was in the macro-state *s*. Thus, higher levels of abstraction screened out all but the most salient positive reward information associated with each state, much as the limbic system is suspected of triaging stimuli based on their associated outcomes (Gruber and McDonald, 2012). This is an important feature for successfully applying the framework to large tasks.

### Hierarchical planning architecture

**Figure 3:**
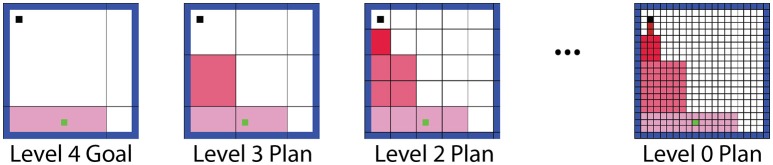
**Illustration of the hierarchical planning process**. If the agent's high-level model prompts the agent to move to the bottom region of the arena, the lower levels perform forward sweeps through the states in their world models, planning a route to this goal at successively finer resolutions.

Our hierarchical framework first selects a goal by identifying the maximum action value available at any level of abstraction. The state (or macro-state) where this action was expected to lead as per the T table was identified as a goal state. The level immediately below the goal level then planned a route from the agent's current location to the goal region. The next level down then refined the first step of this plan at a finer resolution. The first step of the refined plan was refined further by the level below that, and so on until the base (world) level (Figure 3). Thus, the overall process involved successive forward sweeps toward intermediate goals that progressed backward from the high-level goal. These forward sweeps are considered to be functionally similar to the forward sweep phenomena observed in the hippocampus (Johnson and Redish, 2007).

We implemented the planning process using the A* algorithm (Hart et al., 1968)—a heuristic search algorithm which, in this case, was guided by current action value estimates. The A* algorithm performed an efficient forward search through the web of states in the agent's world model to find a path between the current location and the goal region.

An ε-greedy policy operated on the output of this hierarchical planning process. That is, the action specified by the plan was executed with probability ε, and a random action was executed otherwise. Here we used ε = 0.8.

### Context switching algorithm

Switching between different contexts was achieved by comparing a memory of the agent's recent experiences with each of several stored models. A history *H* of the agent's *m* most recent experiences was maintained as the agent traversed the environment. Each experience *e*_*i*_ included an action performed by the agent, and the state transition and reward it caused. The probability *P* (*e*_*i*_) of an experience occurring can be estimated using the lowest-level world model: to do this, we first modify the table R [*s, a, s*′] to track the *distribution* of rewards experienced with a state transition rather than the *average* reward, allowing it to provide an estimate of *P* (*r* | *s, a, s*′) at any time. The probability *P* (*s*′|*s, a*) of the transition itself is then calculated as *P* (*e*_*i*_) = *P* (*s*′|*s, a*) · *P* (*r* | *s, a, s*′), with *P* (*s*′|*s, a*) given in Equation (3).

Assuming that the agent's behavior has already been learned and is not changing with the experiences, the probability of a complete history *H* having occurred under a particular model is:

(5) $$P\left(H\right)=\prod_{i=0}^{m-1}P\left(e_{i}\right)$$

Thus, our framework determined the degree to which each of several stored models explained the experience history, by calculating the probability it assigned to that history. If the model currently in use did not provide the best explanation of the experiences, a different one was selected probabilistically, with a softmax function determining each model's probability of selection based on their likelihood given the experience history. If none of the previously learned models assigned the history a probability above 0.5, or if an expected reward was not obtained, a new model was created to represent the new environment.

### Testing

#### Simulating hippocampal lesions

To test the face validity of our framework as a model of rodent navigation control, we evaluated whether dysfunction of select levels of the agent's spatial hierarchy reproduced behavioral impairments of animals after localized hippocampal damage. Ruediger et al. (2012) found that mice with dorsal hippocampal (dH) lesions showed an impaired ability to find a hidden platform in a water maze task. On the other hand, mice with ventral hippocampal (vH) lesions showed retarded learning, but eventually performed as well as intact animals. We simulated a vH lesion by removing the higher layers from the hierarchy, leaving only the lowest level with the most specific world states (such as would be found in the most dorsal region of the hippocampus). We simulated a dH lesion by removing all but the highest level. We suppose that the low-level world models would be disabled by vH lesions, but that the reinforcement learning mechanism (which likely resides outside the hippocampus; Doya, 2007) would be left intact. Thus, in our simulated dH lesion we replace the lowest-level model-based RL algorithm with model-free RL. We compared agents with simulated lesions to an unimpaired agent in terms of the steps needed to find the goal in a simulated open-field spatial navigation task (Figure 2).

#### Testing adaption to added boundaries

We next sought to test if the hierarchical organization would be advantageous for adapting to sudden changes in the environment. We tested two artificial agents in a changing spatial navigation task: One agent used conventional MBRL (Sutton, 1990); the other used our hierarchical approach. Each agent learned to navigate to a goal in an open arena. After ten trials, boundaries were added to the arena to create one of five new mazes. The agents were forced to adapt to the changes. Agents' performance was measured in terms of the number of steps needed to reach the goal in each trial, and the number of times each agent accessed their world model(s). Model accesses is a proxy to measure the cognitive load imposed on the agent. These simulated mazes measured 16 by 48 discrete states, and the agent was allowed four actions (up, down, left, and right). The agent's state consisted solely of its location in the maze—it could not “see” or otherwise sense barriers or rewards. Attempting to navigate into a wall caused a reward of −1. Finding the goal triggered a reward of 100 and relocated the agent to the start state. Trial length was capped at 5000 steps—upon exceeding this step counts the agent was relocated to the start state and the next trial began. All simulations were repeated 56 times.

The abstraction scheme (Figure 2) and the size of the simulated environments produced six levels of abstraction in the hierarchical model. Model-based RL algorithms at each level were allowed a maximum of 20 model updates per step, for a total of 120. The conventional MBRL algorithm was similarly allowed a maximum of 120 updates per step. Thus, the hierarchical approach employed relaxed model-updating requirements (lighter cognitive load) at low levels of abstraction, being allowed far fewer updates to the environment-level model than the conventional approach (20 vs. 120 updates).

#### Scaling to large problems through hierarchical abstraction

Problems involving many states can be problematic for conventional RL algorithms, because excessive discounting causes reward information to be lost over large distances. The effect is compounded if the agent is allowed finite model updates, and therefore cannot easily propagate reward information across a large model. In contrast, the hierarchical framework may propagate reward information through high-level abstract layers relatively easily, allowing it to scale to large problems.

We tested our framework's ability to solve navigation problems involving many states, by scaling the task in Figure 2 up in size by factors between 1 and 10. We tested the conventional MBRL algorithm and our hierarchical framework in each of these larger mazes, and recorded the number of steps required to reach the goal in each learned solution. The hierarchical and conventional approaches were allowed equal numbers of model updates per step in each test.

## Results

### Simulated hippocampal lesions mimic effects of physical hippocampal lesions

**Figure 4:**
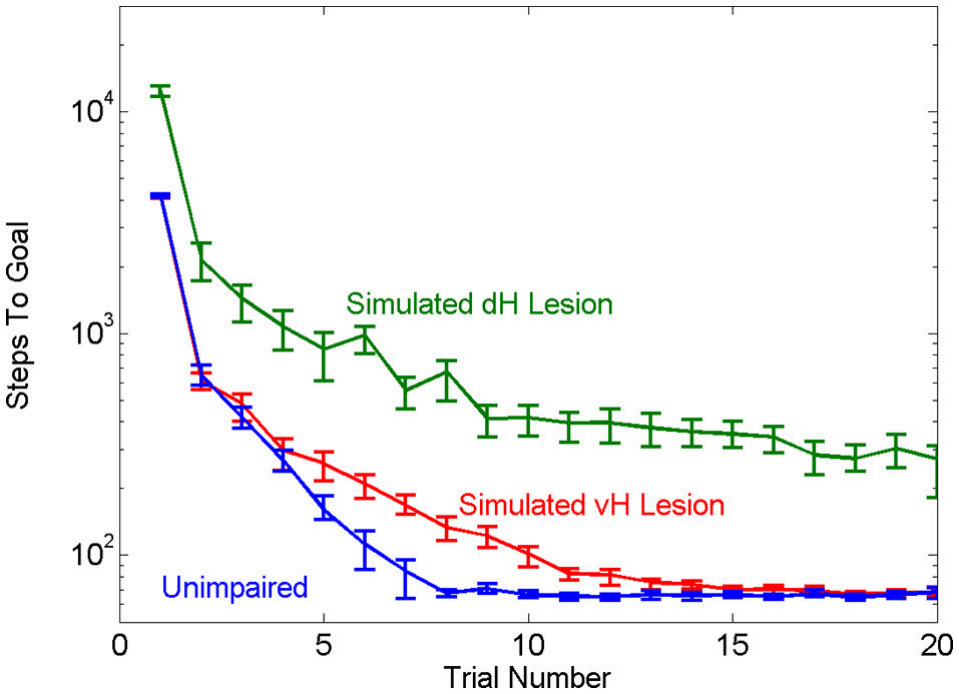
**Effects of simulated vH or dH lesions, in terms of mean number of steps needed for the unimpaired and impaired agents to find the goal in the simple navigation task**. The agent with simulated vH lesion learns the task more slowly than the unimpaired agent, while the agent with simulated dH lesion is impaired across all trials as compared to the other two groups. Error bars show the 95% confidence interval.

Our first objective was to test the face validity of the hierarchical MBRL schema with respect to the navigation properties of mammals. The role of the hippocampus in solving water-maze escape tasks has been studied extensively. In this task, animals are placed into a pool of turbid water in which a small platform is hidden under the surface. Animals are intrinsically motivated to search the pool to find and rest on the platform. Animals quickly acquire the task by learning to swim in a direct path to the platform. Animals with lesions of ventral hippocampus (vH) suffer delayed acquisition, requiring more trials to learn the task, whereas animals with lesions of dorsal hippocampus (dH) never learn to perform the task as well as intact animals (Ruediger et al., 2012). Using our framework to simulate lesions of vH or dH qualitatively replicated these effects of the physical brain lesions. The agent with simulated vH lesion learned the task more slowly than the unimpaired agent but eventually achieved the same asymptotic level of performance. The agent with simulated dH lesion had worse asymptotic performance (Figure 4). The simulated dH lesion impaired the agent's ability to zero-in on the goal; it could use model-based planning at a high level to quickly navigate to the general region of the platform, but once there had to rely on random motion and a slow model-free learning mechanism to reach the goal.

### Efficient spatial navigation and adaptation

**Figure 5:**
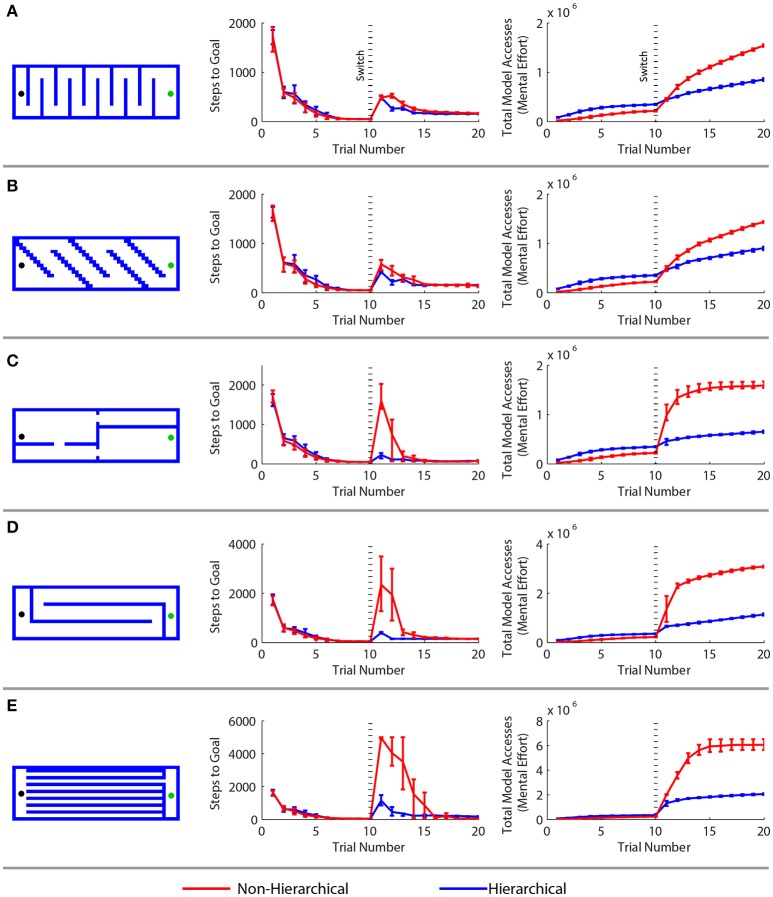
**Comparison of a standard model-based reinforcement learning algorithm with the hierarchical approach in a spatial navigation and adaptation task. (A–E)** Results for five different adaptation tasks. Results are arranged as follows. Left: new maze boundaries that the agents were required to learn after being trained in an open arena for 10 trials. Center: the mean number of steps needed to reach the goal in each trial—the first 10 trials correspond to the open arena, the next 10 to the new maze. Right: the cumulative cognitive effort expended, measured by the mean cumulative sum of model access performed. Error bars show the 2.5 and 97.5 percentile values.

We next investigated whether the spatial abstraction represented in the hierarchy would facilitate adaptation to the sudden addition of obstacles in the environment. To do so, we first trained the hierarchial and conventional agents on an open arena task (as in Figure 2) for 10 trials. After the tenth trial we added boundaries to form one of five different mazes (Figure 5). We computed the number of steps required to reach the goal and the total number of times the agent's model was accessed, a measurements which represents the cumulative sum of mental effort expended during learning. Most of the computational work in model-based RL is in maintenance of the world model: the model must be accessed during the decision making process, and updated after each new experience. The number of model accesses performed by our computational agent serves to represent the mental effort required by the analogous processes in the brain.

The agents performed similarly during the first ten trials in the open arena. The hierarchical approach required more cognitive effort during these trials because of the overhead of maintaining multiple models. However, the hierarchical framework greatly outperformed the conventional algorithm when the agents were forced to adapt to the maze boundaries. In every case, there was a statistically significant (*p* < 0.01 by the Wilcoxon test) difference in the number of steps needed to reach the goal after the change of environment. The hierarchical approach adapted more quickly and used far less computational resources than the standard MBRL algorithm. This is because the boundaries affect low-level navigation, but not the high-level assignment of value to the region of the goal in the environment. At the highest level of abstraction, the task does not change.

**Figure 6:**
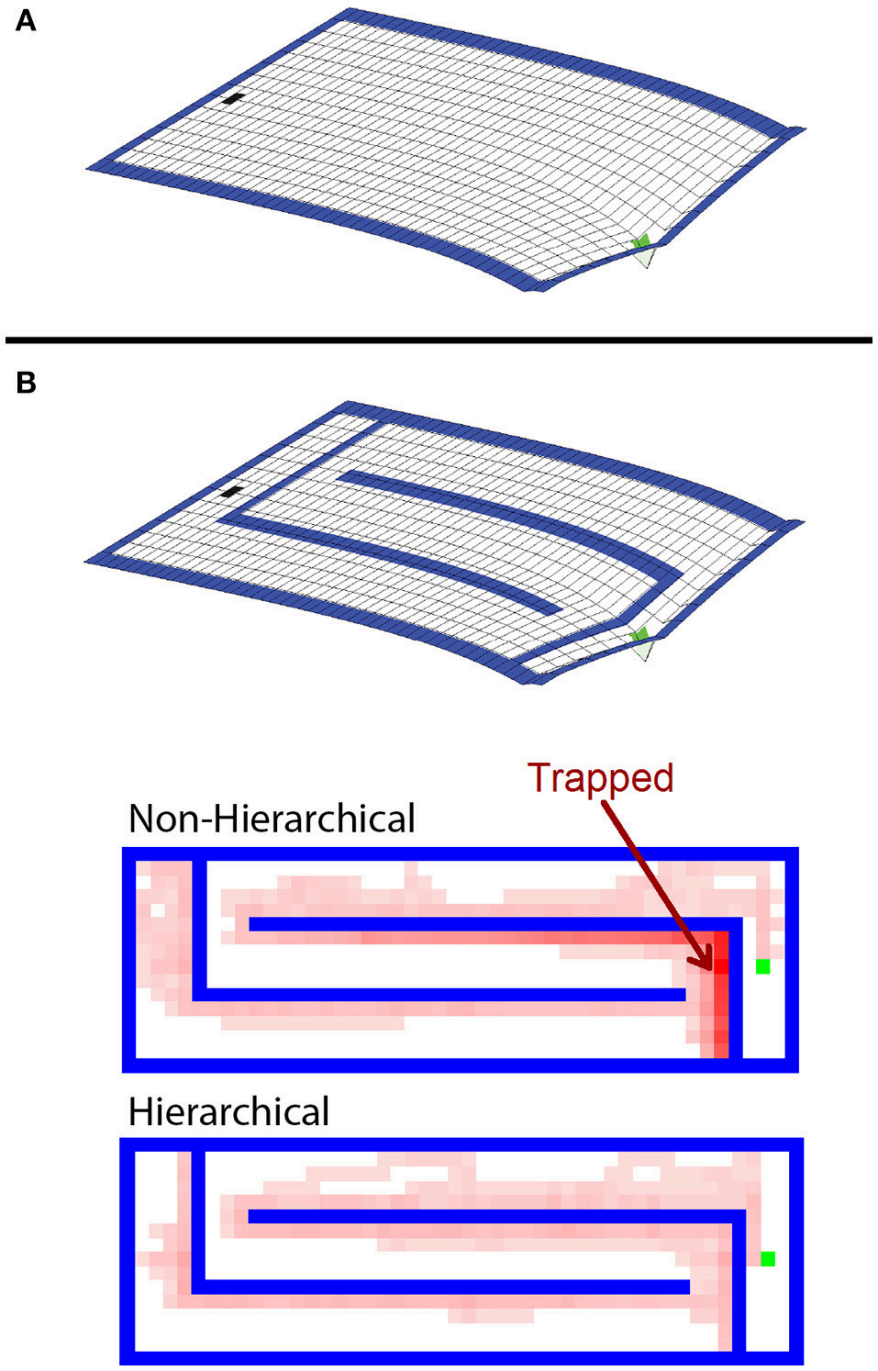
**Analysis of difference in agent flexibility. (A)** Negative of the value gradient learned by the conventional agent in the open arena. After learning, the agent tends to follow this gradient toward the goal from any other state. **(B)** The learned value gradient overlaid with the new maze boundaries, and state occupancy density for trajectories taken by hierarchical and non-hierarchical algorithms during their first four trials in this new environments. Brighter red shading indicates states that were visited with higher relative frequency. The non-hierarchical algorithm is prone to being trapped in areas where the previously learned action values drive it into new boundaries.

RL agents solve navigation tasks by learning a value representation of the states, and ascending the resultant gradient of values to its peak. This can be visualized by inverting the gradient and imagining the agent has a bias to move “downhill” (Figure 6A). The non-hierarchical approach is slow to adapt to the added boundaries because the value gradient learned in the open arena must be drastically altered to reflect the additional maze boundaries, and a conventional MBRL algorithm requires many steps and much computation to make this change. As a result, regions are created where the previously learned action values pin the agent against the new boundaries (Figure 6B), and it becomes temporarily trapped. Further, the more an agent must travel against the learned value gradient, the longer it takes to adapt to the new boundaries. This is why the mazes in Figure 5 with long horizontal runs require the most steps and effort for the conventional MBRL algorithm. In contrast, the hierarchical approach relies primarily on action values at higher levels of abstraction. The lower levels are used primarily for planning rather than representing action values, so the value gradient problem is avoided and the agent can adapt more readily to new boundaries introduced in the previous solution path (Figure 6B). We next demonstrate that the hierarchical framework can also rapidly and efficiently cope with changes in goal location.

### Adapting to changing goals through context switching

The top-down propagation of goal information provides a means of flexibly and efficiently attaining a learned goal when obstacles are introduced. But if the goal itself changes—for instance, if the agent is hungry rather than thirsty, or if the task rules change dramatically—an entirely different model or policy may be necessary. Animals can rapidly switch between learned spatial tasks, and hippocampal damage impairs this flexibility (McDonald et al., 2002). The implementation of MBRL in our framework supports context-dependent policies. If the environment changes or if the current world model no longer adequately explains the agent's experiences in the world, a new context is automatically created (see Section: “Methods”). In the hippocampus, place cells undergo *global remapping* when an environment is switched (Jezek et al., 2011). We propose here that this supports learning of a new model of the world and new policy that does not interfere with previous models. This allows rapid context-driven shifts between world models and policies.

We tested our framework's ability to cope with changing goals through context switching by introducing a new task in which the reward was placed randomly in each trial at one of four locations in an environment measuring 7 by 7 states. We again tested both the new framework and a conventional MBRL algorithm, recording the number of steps needed to reach the goal in each trial. Here the step count was capped at 500 steps.

**Figure 7:**
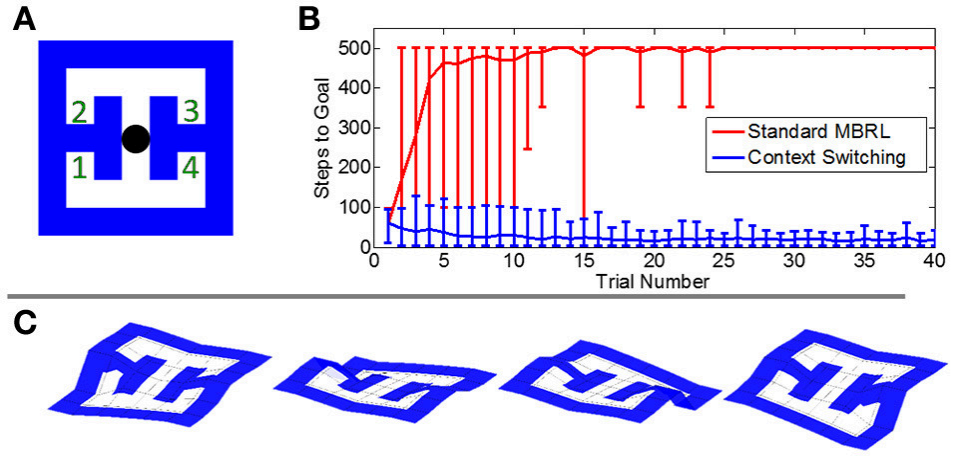
**Comparative performance on a task with probabilistic reward locations. (A)** The simulated environment. Agents began each trial at the black circle. In each trial, a reward was placed randomly at one of the four numbered locations. **(B)** The mean number of steps needed to reach the goal in each trial. The conventional MBRL algorithm failed to learn the task and often did not locate the reward within the maximum number of steps allowed per trial (500). Error bars show the 2.5 and 97.5 percentile values. **(C)** Value gradients in the four contexts learned by the hierarchical framework.

The conventional MBRL algorithm could not solve this task (Figure 7). A failure to acquire reward in a given location persists in the agent's model so that re-exploration of the site is inhibited. In contrast, the new framework's context-switching mechanism allowed it to learn the various reward sites as separate contexts. When the agent arrived at a previously-rewarding location and found no reward, it switched to a context in which the reward is expected elsewhere. Thus, it learned to systematically navigate to each reward site until the goal was found.

### Scaling to large problems

Conventional methods of adapting to environmental change in computational RL include periodically re-exploring previously unrewarding actions (Sutton, 1990; Sutton and Barto, 1998) and *tracking*, in which the agent weights recent experiences more heavily than past ones (Sutton and Barto, 1998) and thus *tracks* the changing solution to a task rather than *converging* on an optimal solution.

Admittedly, these are much simpler methods of adaptation than our hierarchical or context-switching schemes, and in many cases would likely perform as well. However, the hierarchical approach provides an important additional advantage: it can solve large problems more easily than conventional MBRL when the capacity for model updating is finite, because even a very large navigation problem becomes relatively simple at a high level of abstraction. Thus, the hierarchical system may scale to large environments in which finite model updates and excessive discounting over large distances would prevent conventional MBRL from learning useful action values.

This effect is illustrated in Table 1. The hierarchical approach sometimes learns marginally sub-optimal solutions—a trade-off that often accompanies the use of hierarchical abstraction (Hengst, 2011). However the use of hierarchical abstraction allows the agent to solve large navigation problems that the conventional MBRL algorithm cannot. The number of model access required by conventional MBRL appears to be polynomial with respect to the number of states in the maze, while the model accesses required by the hierarchical approach scale linearly. The performance differences between the hierarchical and conventional approaches are statistically significant (*p* < 0.01 by the Wilcoxon test) in all cases except the 4800-state maze, where the conventional agent exhibited extremely inconsistent performance.

**Table 1:** **Number of steps taken and total cumulative model accesses performed during 3 trials in a navigation task (Figure 2) that has been scaled-up in size**.

**States in Maze**	**Non-hierarchical**		**Hierarchical**	
	**Steps Taken (× 1000)**	**Model Accesses (× 1000)**	**Steps Taken (× 1000)**	**Model Accesses (× 1000)**
768	2.99 [2.99–3]	112 [109–114]	1.91 [1.88–1.95]	124 [120–124]
1728	6.67 [6.61–6.81]	313 [274–339]	3.62 [3.23–3.88]	245 [233–261]
3072	11.75 [11.34–12.05]	673 [631–743]	6.9 [6.33–7.45]	510 [455–558]
4800	16.17 [12.18–18.16]	769 [271–1141]	9.74 [9.03–10.63]	758 [701–789]
6912	26.22 [25.54–27.23]	1683 [1310–2101]	13.43 [12.79–14.33]	1061 [1011–1123]
12,288	Failed		25.76 [24.17–27.44]	2185 [2069–2354]
19,200	Failed		36 [34.07–37.95]	3138 [2932–3366]
76,800	Failed		144.78 [136.45–152.32]	14,323 [13,475–15,488]

*Mean and range across 8 repetitions are shown*.

## Discussion

### Contributions of the framework

Foraging and spatial navigation are centrally important for mammalian survival and depend on the hippocampus (Hartley et al., 2003). Our model extends previous RL models by incorporating the spatial abstraction found in the mammalian hippocampus, the concept of forward sweeping search for route-finding, and the concept of context-driven remapping. Where some literature focuses on strict biological accuracy, our implementation of these abstract features has instead focused on testing their computational properties in learning. However, the computational properties explored here generalize to a more biologically accurate setting.

The concepts of hierarchical reinforcement learning and forward sweeping search have been explored individually in existing literature. Hierarchical reinforcement learning has long been a topic of active research, though most algorithmic developments have focused on learning macro-actions, or learning to solve complex problems by first solving simpler sub-problems (Barto and Mahadevan, 2003; Botvinick and Weinstein, 2014). Hinton and Dayan proposed hierarchical abstraction in the state space (Dayan and Hinton, 1993), but their hierarchy enforced strictly top-down control which makes adaptation to change (as in Section: “Efficient Spatial Navigation and Adaptation”) impossible. The use of forward sweeps as part of a process of planning to reach goals has also been investigated (Chersi and Pezzulo, 2012; Erdem and Hasselmo, 2012; Penny et al., 2013), as has the concept of hierarchical selection of goals (Martinet et al., 2011; Cushman and Morris, 2015; Maisto et al., 2015). Our framework provides a novel integration of all these features, yielding a scalable, flexible, and applicable learning framework. It explains animals' ability to learn multiple independent behaviors, adapt quickly to environmental changes, and solve large problems with low cognitive effort. These features of animal behavior cannot be explained or reproduced by conventional MBRL algorithms.

### Hierarchical abstraction in learning

Hierarchical abstraction can make a difficult learning task more tractable. For example, learning to play chess would be extremely difficult if the game were seen as a sequence of individual moves and board positions. There are millions of possible positions after just a few moves, and considering all possibilities is unrealistic. Instead, competent players abstract the game in various ways, considering high-level concepts like the “strength” of one's position. Action selection then becomes a planning process constrained by the high-level goal. This interaction between high-level abstraction and low-level planning provides a means of solving complex problems. In fact, our framework's ability to adapt to environmental change (as illustrated in Section: “Efficient Spatial Navigation and Adaptation”) is actually a by-product of its ability to solve (large) problems through hierarchical abstraction and planning. This ability of our framework partially explains the ability of animals to learn complex tasks, and leads to the prediction that animals with ventral hippocampal damage would be impaired in spatial learning tasks involving very large distances.

Hierarchy and abstraction simplify learning at the expense of the optimality of the learned behavior. The behavior is now merely hierarchically optimal: it is optimal behavior for the abstract task, but likely sub-optimal for the original task (Hengst, 2011). This trade-off is observed in Figure 5E and Table 1, which show the hierarchical approach converging to slightly sub-optimal solutions. Still, biological agents probably rely heavily on this simplicity-optimality trade-off to make learning complex real-world tasks tractable (Botvinick and Weinstein, 2014). Indeed, rats have shown sub-optimal performance as compared to RL algorithms in several tasks (Sul et al., 2011; Skelin et al., 2014).

Our model predicts that higher levels of the hierarchy (vH) are more sensitive to reward outcomes than low levels (dH). Indeed, encoding of reward information in the dH is weak, although some changes to the place fields themselves can occur if reward is consistently associated with one location (Carr and Frank, 2012). Little is known about reward encoding in the vH itself, but a wealth of evidence indicates that its primary limbic and cortical targets express robust reward encoding (Gruber et al., 2010; Euston et al., 2012; Ito and Doya, 2015). Indeed, prevailing theories posit that that neural mechanisms of RL primarily involve other brain structures such as the striatum and neocortex. The striatum and its cortical inputs may also have a hierarchical organization in spatial and non-spatial dimensions (Ito and Doya, 2011) such that the ventral striatum and its inputs from vH and prefrontal cortex represent a high level of abstraction, and the dorsolateral striatum and its inputs from sensory and motor cortices represent low levels (Voorn et al., 2004). Our model thus elegantly fits with proposals that the limbic systems (including vH) are involved in reward processing and goal-directed behavior, while sensorimotor systems are much less sensitive to rewards and implement sensory-response control (Gruber and McDonald, 2012). In this larger context of brain structures, our hierarchical framework provides a novel explanation for why damage to areas such as prefrontal cortex and dorsomedial striatum (involved in higher-level RL layers here) often only cause transient impairments in choice that are noticeable when environmental contingencies change (Robbins, 2007; Castañé et al., 2010). This is because low-level control is sufficient to solve well-learned tasks, while upper levels are engaged when unexpected state transitions occur. This is also consistent with the gradual shift of behavioral control from goal-directed to (lower-level) habitual control (Balleine and O'Doherty, 2010). This has been proposed to be more computationally efficient (Daw et al., 2005), which our data here strongly suggest (Figure 5).

Hierarchical abstraction may be an important part of general *transfer learning*: the ability demonstrated by animals (yet still elusive in artificial intelligence) to apply knowledge learned in one task to solve a different but similar task (Thrun and Pratt, 2012). Moreover, the various forms of hierarchical abstraction may be interrelated as discussed above. One notable non-spatial example comes from Botvinick and Weinstein (2014), who have discussed hierarchical learning of actions, such as learning to perform a complex motor sequence (like grasping an object) as a single macro-action. The question of how an agent learns useful macro-actions remains. Machine learning research has proposed several answers (Stolle and Precup, 2002; Şmşek and Barto, 2004; Taghizadeh and Beigy, 2013), and our hierarchical planning process may hint at another: plans calculated to achieve high-level goals may gradually become macro-actions which an agent can later execute habitually.

### Learning context-dependent policies

Our implementation of multiple context-dependent policies derives from data showing that the ventral hippocampus facilitates learning different policies in different environmental contexts. Specifically, if task demands on a spatial maze are switched, learning the new policy is faster if the hippocampus is intact and the learning takes place in a different room (McDonald et al., 2002). When the intact animal is returned to the original task, it selects between the two learned policies. Our framework posits the explanation that, without the hippocampus-based system for encoding distinct contexts and managing separate models, rats with hippocampal lesions are unable to preserve the first learned model for later retrieval.

Neural activity in the hippocampus responds to minor environmental changes through modulating the activity of the place cells while preserving the spatial encoding (rate remapping; Allen et al., 2012). On the other hand, different spatial contexts are represented by a new set of place cell assignments (global remapping), and the hippocampus can switch rapidly between learned contexts based on cues (Jezek et al., 2011). This could be an important component of contextual control of policies in the brain, which is not present in our current framework. Moreover, the expansion of modalities in the hierarchy beyond physical space (Collin et al., 2015) could account for the critical role of the hippocampus in forward thinking and creativity (Race et al., 2011).

Interestingly, place cell remapping does not occur uniformly along the septotemporal axis of the hippocampus. Changes to the environment or to the task being performed in the environment can induce remapping in the dorsal, but not ventral place fields (Schmidt et al., 2012; Lee et al., 2015; Lu et al., 2015). This contrasts with our context changing mechanism, which always creates an entirely new model at every level. The discrepancy suggests that the brain's mechanism for learning in multiple contexts is more efficient than the mechanism we have implemented here, and is able to transfer some high-level abstract information between contexts. This ability is probably possible in part because spatial representations and value information are stored separately in the hippocampus and striatum, rather than combined as in our abstract framework.

We speculate that our framework could be enhanced by the addition of function of other brain regions. In particular, the prefrontal cortex is strongly implicated in contextual control of behavior and cognitive flexibility (Buckner and Carroll, 2007; Euston et al., 2012). It is very likely that cortex exerts control over the policy, and may do so even if the spatial representation is not globally remapped as we have implemented here.

Another avenue for future development may lie in the more comprehensive and biologically accurate concept of reward being pursued by Gutkin (Keramati and Gutkin, 2011, 2014). While conventional reward-maximizing RL algorithms are based on dopamine-driven learning mechanisms (Doya, 2007) Gutkin proposes an analytical framework in which the objectives of reward maximization and physiological homeostasis coincide, providing a broader view of learning and adaptive behavior. Integration of these ideas with our hierarchical abstraction scheme seems logical and promising.

## Conclusion

While there is much opportunity to expand the computational framework, the present form proposes an interesting relationship between hippocampal place cells and model-based learning mechanisms. The hippocampus' hierarchical representation of space can support a computationally efficient style of learning and adaptation that may not be possible otherwise.

## Author contributions

The work was planned by EC, AL, and AG. Computational work, including algorithm development and implementation, was performed by EC. EC, AL, and AG interpreted test results. The manuscript was written by EC and edited by EC, AL, and AG.

## Funding

This work was supported by the Natural Sciences and Engineering Research Council of Canada (NSERC).

### Conflict of interest statement

The authors declare that the research was conducted in the absence of any commercial or financial relationships that could be construed as a potential conflict of interest.

## References

[B1] Allen K. Rawlins J. N. P. Bannerman D. M. Csicsvari J. (2012). Hippocampal place cells can encode multiple trial-dependent features through rate remapping. J. Neurosci. Off. J. Soc. Neurosci. 32, 14752–14766. 10.1523/JNEUROSCI.6175-11.2012 23077060 PMC3531717

[B2] Balleine B. W. O'Doherty J. P. (2010). Human and rodent homologies in action control: corticostriatal determinants of goal-directed and habitual action. Neuropsychopharmacol. Off. Publ. Am. Coll. Neuropsychopharmacol. 35, 48–69. 10.1038/npp.2009.131 19776734 PMC3055420

[B3] Barto A. G. Mahadevan S. (2003). Recent advances in hierarchical reinforcement learning. Discrete Event Dyn. Syst. 13, 341–379. 10.1023/A:1025696116075

[B4] Botvinick M. Weinstein A. (2014). Model-based hierarchical reinforcement learning and human action control. Philos. Trans. R. Soc. Lond. B Biol. Sci. 369:20130480. 10.1098/rstb.2013.0480 25267822 PMC4186233

[B5] Buckner R. L. Carroll D. C. (2007). Self-projection and the brain. Trends Cogn. Sci. 11, 49–57. 10.1016/j.tics.2006.11.004 17188554

[B6] Carr M. F. Frank L. M. (2012). A single microcircuit with multiple functions: state dependent information processing in the hippocampus. Curr. Opin. Neurobiol. 22, 704–708. 10.1016/j.conb.2012.03.007 22480878 PMC3438355

[B7] Castañé A. Theobald D. E. H. Robbins T. W. (2010). Selective lesions of the dorsomedial striatum impair serial spatial reversal learning in rats. Behav. Brain Res. 210, 74–83. 10.1016/j.bbr.2010.02.017 20153781 PMC3038258

[B8] Chersi F. Pezzulo G. (2012). Using hippocampal-striatal loops for spatial navigation and goal-directed decision-making. Cogn. Process. 13 (Suppl. 1), S125–S129. 10.1007/s10339-012-0475-7 22806662

[B9] Collin S. H. P. Milivojevic B. Doeller C. F. (2015). Memory hierarchies map onto the hippocampal long axis in humans. Nat. Neurosci. 18, 1562–1564. 10.1038/nn.4138 26479587 PMC4665212

[B10] Cushman F. Morris A. (2015). Habitual control of goal selection in humans. Proc. Natl. Acad. Sci. U.S.A. 112, 13817–13822. 10.1073/pnas.1506367112 26460050 PMC4653221

[B11] Daw N. D. (2012). Model-based reinforcement learning as cognitive search neurocomputational theories, in Cognitive Search: Evolution, Algorithms, and the Brain, eds Todd P. M. Hills T. T. Robbins T. W. (Cambridge: IEEE; MIT Press), 195–207.

[B12] Daw N. D. Niv Y. Dayan P. (2005). Uncertainty-based competition between prefrontal and dorsolateral striatal systems for behavioral control. Nat. Neurosci. 8, 1704–1711. 10.1038/nn1560 16286932

[B13] Daw N. D. O'Doherty J. P. Dayan P. Seymour B. Dolan R. J. (2006). Cortical substrates for exploratory decisions in humans. Nature 441, 876–879. 10.1038/nature04766 16778890 PMC2635947

[B14] Dayan P. Hinton G. E. (1993). Feudal reinforcement learning, in Advances in Neural Information Processing Systems, eds Hanson S. J. Cowan J. D. Giles C. L. (Cambridge: Morgan Kaufmann Publishers; MIT Press), 271–271.

[B15] Doya K. (2007). Reinforcement learning: computational theory and biological mechanisms. HFSP J. 1, 30–40. 10.2976/1.2732246 19404458 PMC2645553

[B16] Erdem U. M. Hasselmo M. (2012). A goal-directed spatial navigation model using forward trajectory planning based on grid cells. Eur. J. Neurosci. 35, 916–931. 10.1111/j.1460-9568.2012.08015.x 22393918 PMC3564559

[B17] Euston D. R. Gruber A. J. McNaughton B. L. (2012). The role of medial prefrontal cortex in memory and decision making. Neuron 76, 1057–1070. 10.1016/j.neuron.2012.12.002 23259943 PMC3562704

[B18] Gruber A. J. Calhoon G. G. Shusterman I. Schoenbaum G. Roesch M. R. O'Donnell P. (2010). More is less: a disinhibited prefrontal cortex impairs cognitive flexibility. J. Neurosci. Off. J. Soc. Neurosci. 30, 17102–17110. 10.1523/JNEUROSCI.4623-10.2010 21159980 PMC3073623

[B19] Gruber A. J. McDonald R. J. (2012). Context, emotion, and the strategic pursuit of goals: interactions among multiple brain systems controlling motivated behavior. Front. Behav. Neurosci. 6:50. 10.3389/fnbeh.2012.00050 22876225 PMC3411069

[B20] Hart P. E. Nilsson N. J. Raphael B. (1968). A formal basis for the heuristic determination of minimum cost paths. IEEE Trans. Syst. Sci. Cybern. 4, 100–107. 10.1109/TSSC.1968.300136

[B21] Hartley T. Maguire E. A. Spiers H. J. Burgess N. (2003). The well-worn route and the path less traveled: distinct neural bases of route following and wayfinding in humans. Neuron 37, 877–888. 10.1016/S0896-6273(03)00095-3 12628177

[B22] Hengst B. (2011). Hierarchical reinforcement learning, in Encyclopedia of Machine Learning, eds Sammut C. Webb G. I. (New York, NY: Springer), 495–502.

[B23] Ito M. Doya K. (2011). Multiple representations and algorithms for reinforcement learning in the cortico-basal ganglia circuit. Curr. Opin. Neurobiol. 21, 368–373. 10.1016/j.conb.2011.04.001 21531544

[B24] Ito M. Doya K. (2015). Distinct neural representation in the dorsolateral, dorsomedial, and ventral parts of the striatum during fixed- and free-choice tasks. J. Neurosci. 35, 3499–3514. 10.1523/JNEUROSCI.1962-14.2015 25716849 PMC4339358

[B25] Jezek K. Henriksen E. J. Treves A. Moser E. I. Moser M.-B. (2011). Theta-paced flickering between place-cell maps in the hippocampus. Nature 478, 246–249. 10.1038/nature10439 21964339

[B26] Johnson A. Redish A. D. (2007). Neural Ensembles in CA3 Transiently Encode Paths Forward of the Animal at a Decision Point. J. Neurosci. 27, 12176–12189. 10.1523/JNEUROSCI.3761-07.2007 17989284 PMC6673267

[B27] Keinath A. T. Wang M. E. Wann E. G. Yuan R. K. Dudman J. T. Muzzio I. A. (2014). Precise spatial coding is preserved along the longitudinal hippocampal axis. Hippocampus 24, 1533–1548. 10.1002/hipo.22333 25045084 PMC4447627

[B28] Keramati M. Gutkin B. (2014). Homeostatic reinforcement learning for integrating reward collection and physiological stability. eLife 3:e04811. 10.7554/eLife.04811 25457346 PMC4270100

[B29] Keramati M. Gutkin B. S. (2011). A reinforcement learning theory for homeostatic regulation, in Advances in Neural Information Processing Systems, Vol. 24, eds Shawe-Taylor J. Zemel R. S. Bartlett P. L. Pereira F. Weinberger K. Q. (Curran Associates, Inc), 82–90. Available online at: http://papers.nips.cc/paper/4437-a-reinforcement-learning-theory-for-homeostatic-regulation.pdf (Accessed November 11, 2016)

[B30] Lee H. Wang C. Deshmukh S. S. Knierim J. J. (2015). Neural population evidence of functional heterogeneity along the CA3 transverse axis: pattern completion versus pattern separation. Neuron 87, 1093–1105. 10.1016/j.neuron.2015.07.012 26298276 PMC4548827

[B31] Lu L. Igarashi K. M. Witter M. P. Moser E. I. Moser M.-B. (2015). Topography of Place Maps along the CA3-to-CA2 Axis of the Hippocampus. Neuron 87, 1078–1092. 10.1016/j.neuron.2015.07.007 26298277

[B32] Maisto D. Donnarumma F. Pezzulo G. (2015). Divide et impera: subgoaling reduces the complexity of probabilistic inference and problem solving. J. R. Soc. Interface 12:20141335. 10.1098/rsif.2014.1335 25652466 PMC4345499

[B33] Martinet L.-E. Sheynikhovich D. Benchenane K. Arleo A. (2011). Spatial learning and action planning in a prefrontal cortical network model. PLoS Comput. Biol. 7:e1002045. 10.1371/journal.pcbi.1002045 21625569 PMC3098199

[B34] McDonald R. J. Ko C. H. Hong N. S. (2002). Attenuation of context-specific inhibition on reversal learning of a stimulus-response task in rats with neurotoxic hippocampal damage. Behav. Brain Res. 136, 113–126. 10.1016/S0166-4328(02)00104-3 12385796

[B35] Montague P. R. Dayan P. Sejnowski T. J. (1996). A framework for mesencephalic dopamine systems based on predictive Hebbian learning. J. Neurosci. 16, 1936–1947. 8774460 10.1523/JNEUROSCI.16-05-01936.1996 PMC6578666

[B36] Moore A. W. Atkeson C. G. (1993). Prioritized sweeping: Reinforcement learning with less data and less time. Mach. Learn. 13, 103–130. 10.1007/BF00993104

[B37] Moser M.-B. Rowland D. C. Moser E. I. (2015). Place Cells, Grid Cells, and Memory. Cold Spring Harb. Perspect. Biol. 7:a021808. 10.1101/cshperspect.a021808 25646382 PMC4315928

[B38] Packard M. G. McGaugh J. L. (1996). Inactivation of hippocampus or caudate nucleus with lidocaine differentially affects expression of place and response learning. Neurobiol. Learn. Mem. 65, 65–72. 10.1006/nlme.1996.0007 8673408

[B39] Passingham R. E. Wise S. P. (2014). The Neurobiology of the Prefrontal Cortex: Anatomy, Evolution, and the Origin of Insight. Oxford: Oxford University Press.

[B40] Penny W. D. Zeidman P. Burgess N. (2013). Forward and backward inference in spatial cognition. PLoS Comput. Biol. 9:e1003383. 10.1371/journal.pcbi.1003383 24348230 PMC3861045

[B41] Pfeiffer B. E. Foster D. J. (2013). Hippocampal place-cell sequences depict future paths to remembered goals. Nature 497, 74–79. 10.1038/nature12112 23594744 PMC3990408

[B42] Race E. Keane M. M. Verfaellie M. (2011). Medial temporal lobe damage causes deficits in episodic memory and episodic future thinking not attributable to deficits in narrative construction. J. Neurosci. 31, 10262–10269. 10.1523/JNEUROSCI.1145-11.2011 21753003 PMC4539132

[B43] Reynolds J. N. Hyland B. I. Wickens J. R. (2001). A cellular mechanism of reward-related learning. Nature 413, 67–70. 10.1038/35092560 11544526

[B44] Robbins T. (2007). Shifting and stopping: fronto-striatal substrates, neurochemical modulation and clinical implications. Philos. Trans. R. Soc. B Biol. Sci. 362, 917–932. 10.1098/rstb.2007.2097 17412678 PMC2430006

[B45] Roberts W. A. Cruz C. Tremblay J. (2007). Rats take correct novel routes and shortcuts in an enclosed maze. J. Exp. Psychol. Anim. Behav. Process. 33, 79–91. 10.1037/0097-7403.33.2.79 17469957

[B46] Ruediger S. Spirig D. Donato F. Caroni P. (2012). Goal-oriented searching mediated by ventral hippocampus early in trial-and-error learning. Nat. Neurosci. 15, 1563–1571. 10.1038/nn.3224 23001061

[B47] Samejima K. Ueda Y. Doya K. Kimura M. (2005). Representation of action-specific reward values in the striatum. Science 310, 1337–1340. 10.1126/science.1115270 16311337

[B48] Schmidt B. Satvat E. Argraves M. Markus E. J. Marrone D. F. (2012). Cognitive demands induce selective hippocampal reorganization: *Arc* expression in a place and response task. Hippocampus 22, 2114–2126. 10.1002/hipo.22031 22573703

[B49] Seo H. Cai X. Donahue C. H. Lee D. (2014). Neural correlates of strategic reasoning during competitive games. Science 346, 340–343. 10.1126/science.1256254 25236468 PMC4201877

[B50] Singer A. C. Frank L. M. (2009). Rewarded outcomes enhance reactivation of experience in the Hippocampus. Neuron 64, 910–921. 10.1016/j.neuron.2009.11.016 20064396 PMC2807414

[B51] Skelin I. Hakstol R. VanOyen J. Mudiayi D. Molina L. A. Holec V.. (2014). Lesions of dorsal striatum eliminate lose-switch responding but not mixed-response strategies in rats. Eur. J. Neurosci. 39, 1655–1663. 10.1111/ejn.12518 24602013

[B52] Şmşek Ö. Barto A. G. (2004). Using relative novelty to identify useful temporal abstractions in reinforcement learning, in Proceedings of the Twenty-First International Conference on Machine Learning (ACM), 95 Available online at: http://dl.acm.org/citation.cfm?id=1015353 (Accessed September 8, 2015).

[B53] Stolle M. Precup D. (2002). Learning options in reinforcement learning, in Lecture Notes in Computer Science, eds Koenig S. Holte R. C. (Berlin; Heidelberg: Springer), 212–223.

[B54] Strange B. A. Witter M. P. Lein E. S. Moser E. I. (2014). Functional organization of the hippocampal longitudinal axis. Nat. Rev. Neurosci. 15, 655–669. 10.1038/nrn3785 25234264

[B55] Sul J. H. Jo S. Lee D. Jung M. W. (2011). Role of rodent secondary motor cortex in value-based action selection. Nat. Neurosci. 14, 1202–1208. 10.1038/nn.2881 21841777 PMC3164897

[B56] Sutton R. S. (1990). Integrated architectures for learning, planning, and reacting based on approximating dynamic programming, in Proceedings of the Seventh International Conference on Machine Learning (New York, NY), 216–224.

[B57] Sutton R. S. Barto A. G. (1998). Reinforcement Learning: An Introduction. MIT Press Cambridge Available online at: http://www.cell.com/trends/cognitive-sciences/pdf/S1364-6613(99)01331-5.pdf (Accessed December 17, 2015).

[B58] Taghizadeh N. Beigy H. (2013). A novel graphical approach to automatic abstraction in reinforcement learning. Robot. Auton. Syst. 61, 821–835. 10.1016/j.robot.2013.04.010

[B59] Thrun S. Pratt L. (2012). Learning to Learn. Norwell, MA: Springer Science & Business Media.

[B60] Voorn P. Vanderschuren L. J. M. J. Groenewegen H. J. Robbins T. W. Pennartz C. M. A. (2004). Putting a spin on the dorsal-ventral divide of the striatum. Trends Neurosci. 27, 468–474. 10.1016/j.tins.2004.06.006 15271494

[B61] Wilson M. A. McNaughton B. L. (1994). Reactivation of hippocampal ensemble memories during sleep. Science 265, 676–679. 8036517 10.1126/science.8036517

